# 3D sub-pixel correlation length imaging

**DOI:** 10.1038/s41598-020-57988-7

**Published:** 2020-01-22

**Authors:** R. P. Harti, M. Strobl, J. Valsecchi, J. Hovind, C. Grünzweig

**Affiliations:** 1 Laboratory for Neutron Scattering and Imaging, Paul Scherrer Institut, Switzerland; 2 Niels Bohr Institute, Copenhagen, Denmark; 3 Design and Disruptive Technologies, Leica Geosystems, AG Switzerland

**Keywords:** Structure of solids and liquids, Topological matter

## Abstract

Quantitative 2D neutron dark-field-imaging with neutron grating interferometry has been used to characterize structures in the size range below the imaging resolution. We present the first 3D quantitative neutron dark-field imaging experiment. We characterize sub-pixel structure sizes below the imaging resolution in tomography by quantitatively analyzing the change in dark-field contrast with varying neutron wavelength. This proof of principle experiment uses a dedicated reference sample with four different solutions of microspheres, each with a different diameter. The result is a 3D tomogram featuring a real space scattering function in each voxel. The presented experiment is expected to mark the path for future material science research through the individual quantification of small-angle scattering structures in each voxel of a volume of a bulk inhomogeneous sample material.

## Introduction

Dark-field imaging (DFI) with neutron grating interferometers is often used as a qualitative method to image the distribution of scattering structures in a sample. This way structures like magnetic domain walls^1–7^ or microstructural defects can be located within a sample^8–11^. Recently quantification was enabled through theoretical^12^ and experimental developments^13^. The setups used for either of these experiments were based on the Talbot-Lau geometry with three gratings^14^. Two absorption gratings referred to as G0 and G2 as well as one phase grating G1.

Quantitative DFI in 2D was presented by wavelength as well as sample to grating distance variation^13,15^ All DFI imaging is based on the variation of the setup dependent autocorrelation length ξ defined as^12^.

1 $${\rm{\xi }}=\frac{\lambda {L}_{s}}{{p}_{2}}$$

with λ being the neutron wavelength, L_s_ the sample to G2 grating distance and p_2_ the period of G2. Some of the previously presented work used specialized setups to increase the distance between G1 and G2 in order to being able to position the sample between G1 and G2. The setup used for 3D quantitative dark-field imaging presented here resembles a Talbot-Lau interferometer at the first Talbot distance.

**Figure 1:**
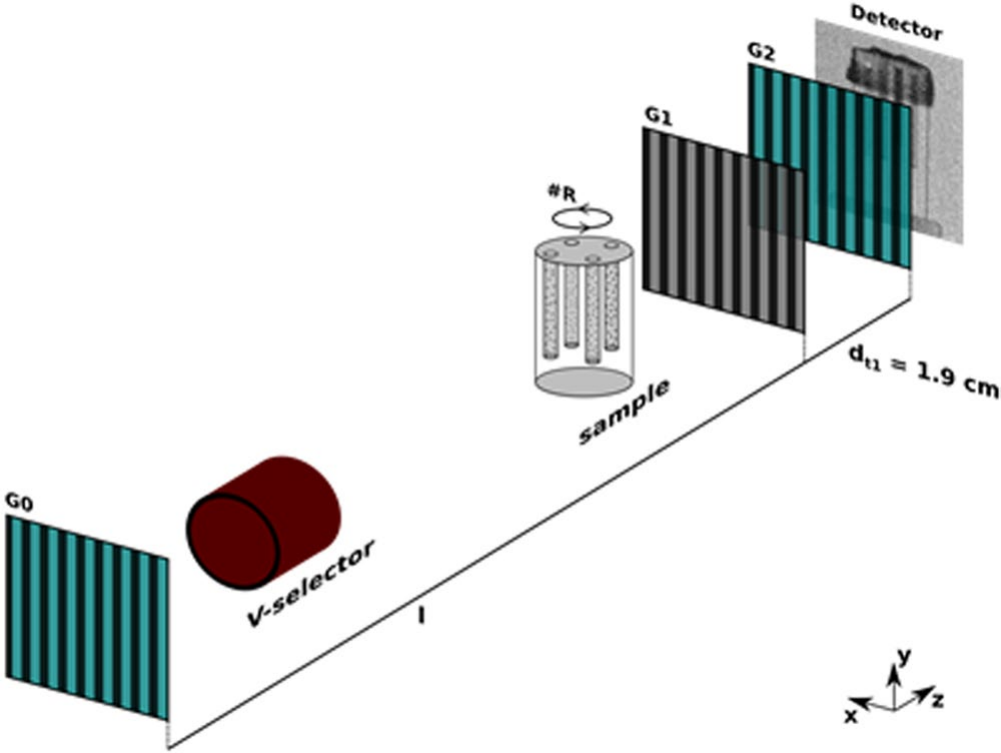
nGI setup for a DFI tomography experiment in the first Talbot order. The sample is positioned between G0 and G1 and can be rotated around the y-axis at rotation positions #R to facilitate tomography experiments. The detector shows a DFI projection as example.

The detailed parameters of the setup, as well as the accessible ξ range, through variation of λ are presented in Table 1. Utilizing a first Talbot order setup the distance between G1 and G2 is fairly short with 19.4 mm. On the other hand, the G0 to G1 distance is very large and was set to 5.23 m. Figure 1 illustrates the setup and indicates the sample position between G0 and G1. The sample would not fit between G1 and G2 in our measurement, nor would the distance L_s_ be sufficiently well defined. In our measurements the sample is placed upstream of G1, in which case L_s_ in the equation describing the probed correlation length ξ is now described by the effective distance^12^.

2 $${L^{\prime} }_{s}=\frac{(l+d-{L}_{s})d}{l}$$

**Table 1:** Setup details for the nGI used during tomographic experiment.

d_tn_	p_0_ [μm]	p_1_ [μm]	p_2_ [μm]	l [m]	d [cm]	ξ scan parameter	ξ range [μm]
1	1076	7.97	4	5.23	1.9	λ	1.5–2.5

d_tn_: Talbot order; p_0_: Period G0; p_1_: Period G1; p_2_: Period G2; l: Distance G0 - G1; d: Distance G1 - G2.

Thus, the variation of this length and therefore ξ for the sample extension along the beam direction is negligible and does not affect the tomographic approach taken here.

The design wavelength of the setup is set at 4.1 Å, which was selected using the velocity selector available at the ICON imaging instrument of the Paul Scherrer Institute^16^. While the grating interferometer setup has a design wavelength, we will use a change in wavelength to scan the autocorrelation length of the setup and thus extract the projected density correlation function in each voxel of the tomogram. The voxel size of the final tomograms was 90 μm. The corresponding procedure was first introduced for two-dimensional studies in^13^.

**Figure 2:**
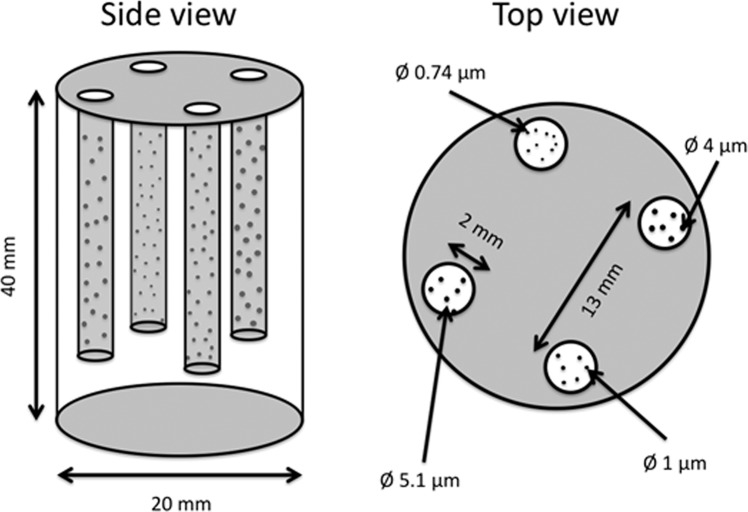
Schematic drawing of the dedicated sample container made from aluminum for tomographic dark-field imaging. Four cylindrical holes with a diameter of 2 mm are embedded into a larger cylinder with a diameter of 20 mm. The four small cylindrical holes contain a low concentration solution of polystyrene microspheres in H_2_O/D_2_O solution with different diameters each as indicated in the top view.

Similar to the work in 2D we chose low concentration solutions of polystyrene micro particles as a reference sample material. Figure 2 shows a schematic drawing of the dedicated sample container designed for the measurement. It is an aluminum cylinder with a diameter of 20 mm in which four cylindrical holes with diameters of 2 mm have been drilled. The cylinder has a height of 40 mm and thus fits within the field of view of the grating interferometer.

The sample container allows for four different solutions of micro particles to be filled in the cylindrical holes (Fig. 2). We used an H_2_O/D_2_O mixture with a ratio of 59:41 to create solutions with concentration 8.55 wt% of polystyrene micro particles. The H_2_O/D_2_O ratio was determined such that it has the same mass density as the polystyrene particles to prevent sedimentation. Four solutions have been prepared containing particles with diameters of 0.74 μm, 1 μm, 4 μm and 5.1 μm. These particle diameters were chosen to represent structures below and above the correlation length range assessable with our setup. Considering the change in DFI value for increasing ξ values, we expect to have a constant DFI value for solutions with particles smaller than the scanning range and an increasing contrast for particles larger than the scanning range.

The sample parameters were chosen in order to prevent the DFI signal from reaching saturation, i.e. total extinction of local visibility of the interference pattern, at any projection during the rotation and wavelength scan. We estimated the expected DFI signal from previous 2-dimensional studies^13^ and positioned the holes in the cylinder such to not cause more than two cylindrical holes to overlap at any given projection.

### Experimental data acquisition

The data acquisition procedure for a DFI tomography is significantly different from the procedure used in conventional transmission tomography. The reason for this is that each projection in grating interferometry is composed of a series of images due to the required step scan of a grating. Thus, the data acquisition times are significantly longer than conventional experiments and the acquisition routine has to be adapted to record both the rotation necessary for a tomography and the grating stepping necessary for DFI.

In addition to the considerations of sample rotation and grating stepping the extraction of the projected density correlation function from DFIs requires a variation in ξ. For the tomography experiment, we realized this variation through changing the neutron wavelength using the velocity selector at the ICON beamline at PSI to consecutively provide different wavelengths within the range from 3 to 5 Å.

**Figure 3:**
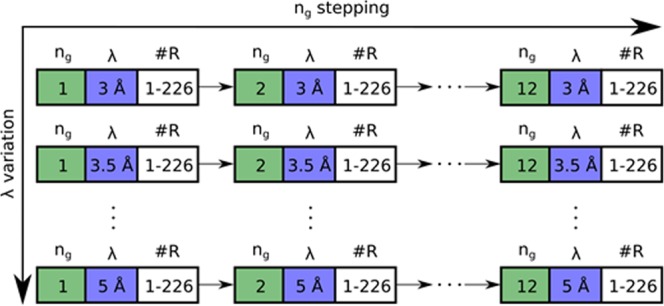
Data recording procedure for multiple ξ DFI tomography. The y-axis represents a λ variation, for λ = 3, 3.5, 4, 4.5, 5 Å, while keeping the grating position n_g_ constant. The x-axis represents an n_g_ variation with constant λ, for n_g_ = 1, 2, 3,…,12. At each position, a tomographic dataset was recorded by rotating the sample 360 degrees with 226 rotation positions #R.

Figure 3 illustrates the data acquisition procedure. First, at each setting of the grating scan and the wavelength, a full tomographic scan with 226 projections covering 360 degrees of sample rotation was performed. Then the wavelength λ was changed and another tomographic scan performed. After performing tomographies for all programmed wavelengths, the grating was stepped to *n*_*g*_ = 2 and the full tomography acquisition procedure was repeated until the process was finished at the final *n*_*g*_ = 12. Thus, each individual DFI tomography is composed of 2712 images with an exposure time of 10 seconds each, resulting in a total exposure time for an individual DFI tomography of around 7.5 hours excluding motor movements. To extract the projected density correlation function in each voxel, we recorded tomographies at neutron wavelengths λ of 3 Å, 3.5 Å, 4 Å, 4.5 Å and 5 Å, resulting in measurements at ξ values of 1.5 μm, 1.75 μm, 2.00 μm, 2.25 μm and 2.5 μm. Thus, the total exposure time for the projections of the tomography experiment was slightly more than 1.5 days. Additionally, the time for motor movements and the recording of open beams has to be considered, making 3D quantitative dark-field imaging a time expensive neutron imaging experiment.

### Data reduction

**Figure 4:**
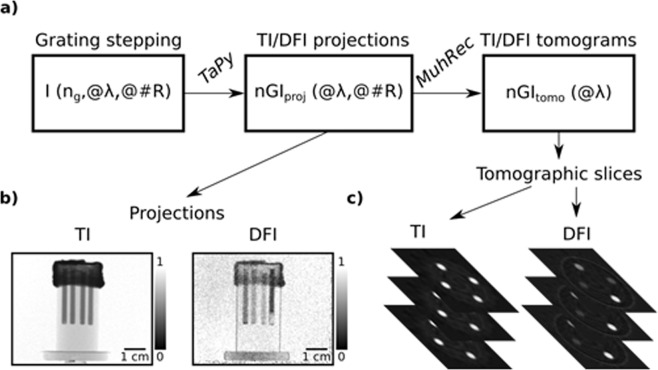
Multi wavelength DFI data reduction procedure for DFI tomography. (**a**) illustrates the path from individual images I with a variation of the grating position n_g_ at fixed wavelength λ and rotation position #R to an nGI dataset at fixed λ and #R, composed of TI and DFI projections using TaPy^17^. These projections were then used to generate DFI and TI tomograms nGI_tomo_ at fixed wavelengths λ. (**b**) exemplary presentation of a TI and an DFI projection and (**c**) represents the tomograms through selected tomographic slices^20,21^.

The data reduction of the DFIs was performed using the *TaPy* software^17^. Figure 4a) illustrates the data reduction workflow for the quantitative DFI tomography. The grating stepping images *I(n*_*g*_*,@*λ*, @#R)* for *n*_*g*_ = 1.0.12 at fixed λ and *#R* are processed using *TaPy*. The matrix implementation of the data reduction algorithm for complex batches described in^18^, enabled to produce transmission images (TIs) and dark-field image (DFIs) for all individual wavelengths λ and projections *#R* in one automated process. An example of individual TI and DFI in a single projection is shown in Fig. 4b. Both the TIs and DFIs corresponding to an individual wavelength were used for conventional tomographic reconstruction with the in-house developed software *MuhRec*^16,19^. The tomographic reconstruction in *MuhRec* results in TI and DFI tomograms nGI_tomo_(@λ) for each wavelength. Consequently, five individual dark-field tomographies at five different wavelengths, i.e. correlation lengths ξ have been produced. Corresponding image data is presented in the form of individual cross sections, i.e. as tomographic slices, as illustrated in Fig. 4c.

**Figure 5:**
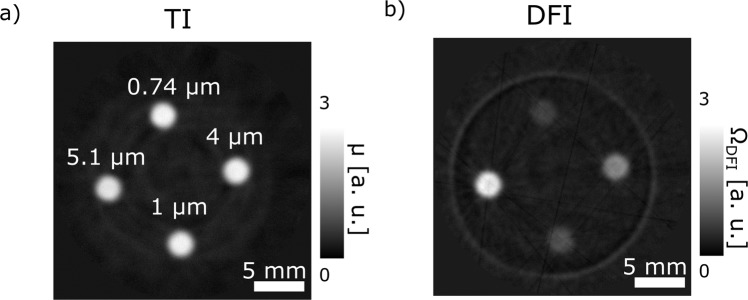
Comparison of a TI and DFI slice from the tomographies. (**a**) An individual slice, out of 530, of the tomographic dataset reconstructed from the TI images at 3.5 Å, representing the attenuation coefficient value distribution of the sample. The four cylindrical holes with the micro particles in H_2_O/D_2_O solution do all exhibit the same signal. (**b**) The same slice as (**a**), but reconstructed from the DFI dataset at 3.5 Å. Each micro particle solution shows different scattering strength Ω, related to the diameter of the micro particles in solution.

Figure 5 presents a comparison of an individual slice of the tomography of the sample at a neutron wavelength of 3.5 Å for the transmission and dark-field signal. The TI in Fig. 5a shows no difference in attenuation coefficient for the different solutions of micro particles in the holes of the Al matrix. This is to be expected due to the fact that all holes contain samples of 8.55 wt% polystyrene particles in the same H_2_O/D_2_O solution. Thus all have the same attenuation coefficient as reflected in the TIs. In contrast, the DFI in Fig. 5b shows clear differences between the individual samples in the holes. Depending on the radius of the polystyrene particles, the signal differs significantly, despite all of them containing the same concentration of polystyrene.

## Data analyses

**Figure 6:**
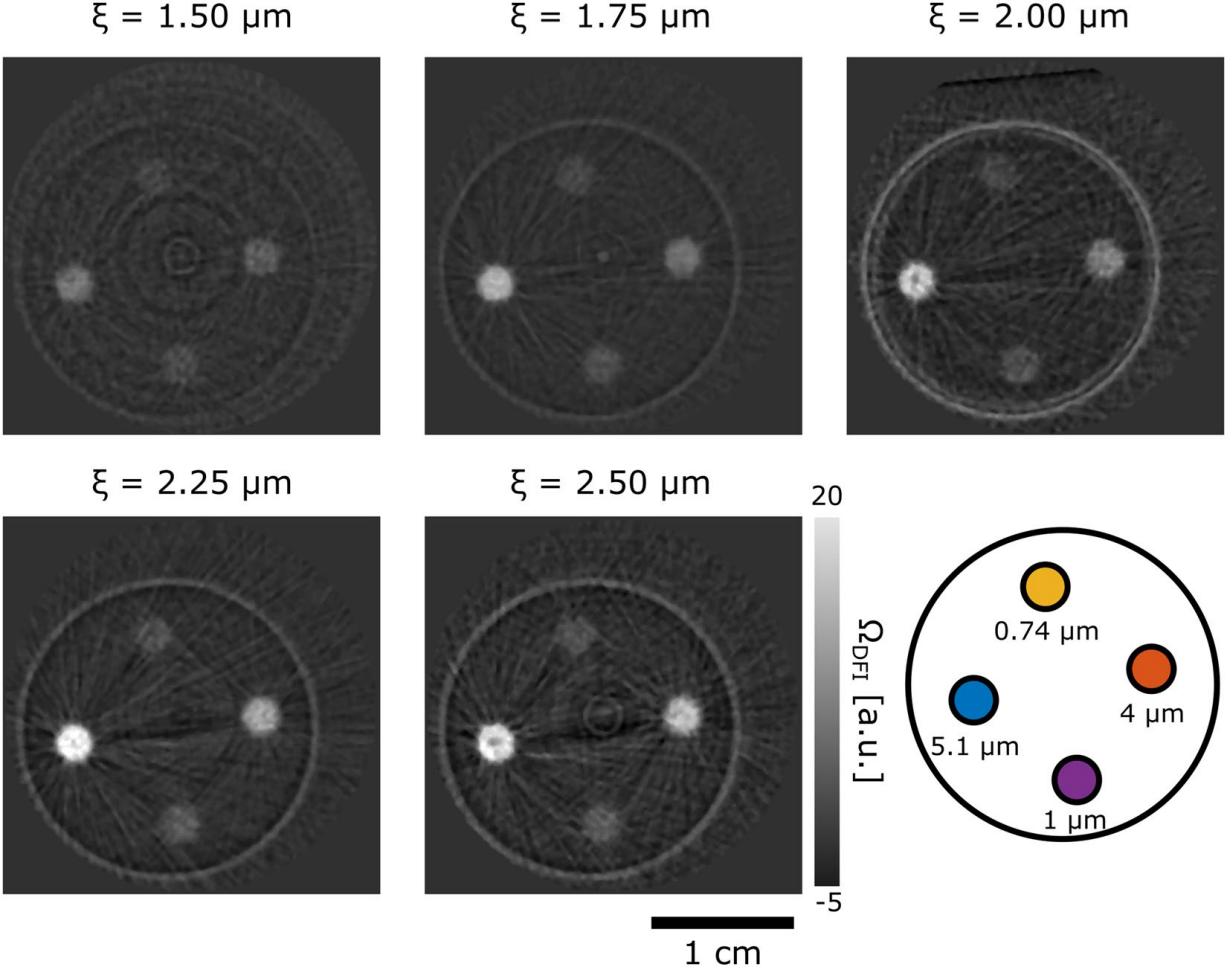
DFI tomography of specimen containing volumes of diluted micro particles. Corresponding individual slices through reconstructed tomographic volumes for scans at varying probed correlation length ξ, i.e. wavelengths of 3 Å, 3.5 Å, 4 Å, 4.5 Å and 5 Å. Each of the four cross sections represents a cut through the cylindrical sample matrix containing four solutions of different polystyrene particles with diameters of 0.74 μm, 1 μm, 4 μm and 5.1 μm, respectively. The schematic drawing illustrates the specific locations of the different micro particle solutions and the color-code corresponds to the plot in Fig. 7.

The tomographic nature of the data allows us to extract values individually for every voxel of the reconstructed volume. Figure 6 shows individual crossectional slices of the reconstructed tomograms for different wavelengths, thus for varying ξ values, which are indicated above the images. The values, in particular in positions of significant small angle scattering are positive, due to the fact that the reconstruction algorithm program for attenuation data was utilized, which returns the attenuation coefficient μ in standard units of [cm^−1^]. These are computed from measured data of the form

3 $$T{I}_{\theta }={I}_{\theta }/{I}_{0}={e}^{-{\int }^{}\mu (x,y,z)ds}$$

In analogy the DFI values can be described by^12^

4 $$DF{I}_{\theta }(\xi )={V}_{\theta }/{V}_{0}={e}^{-{\int }^{}{\Omega }_{DFI}(x,y,z)ds}={e}^{{\int }^{}\Sigma (x,y,z)(G(\xi ,x,y,z)-1){\rm{ds}}}$$

where V is the measured local grating interferometer visibility and the subscripts 0 and θ refer to open beam and sample projection angle and *s* denotes the beam path. The exponent on the right hand side of Eq.(4) does not contain a minus sign, but becomes negative due to the fact that G ≤ 1, in particular G < 1 for ξ  > 0. Utilizing the conventional reconstruction thus returns the DFI signal with the negative exponent Ω_DFI_ = −Σ(G − 1) = Σ(1 − G) [cm^−1^]. This can serve as a measure of the small-angle scattering strength at the particular wavelengths utilized. However, the scattering strength and hence the contrast increase with increasing wavelength as the small-angle scattering probability is

5 $$\Sigma ={\lambda }^{2}{(\Delta \rho )}^{2}\phi (1-\phi ){\rm{\chi }}$$

where Δ*ρ* is the scattering length density contrast between particles and solution, *ϕ* is the volume fraction of the particles and χ being the characteristic structure size of the scattering inhomogenities, here the radius of the spherical particles. Since the variation in ξ was achieved by the variation of the neutron wavelength the tomographies have to be corrected for the wavelength dependence of the macroscopic scattering cross-section in order to extract structural information in terms of the projected density correlation function G(ξ) and the structure size χ independent of measurement characteristics. Thus, the reconstructed DFI values in the tomograms have been normalized as expressed by

6 $$-{\Omega }_{{\rm{DFI}}}/{\lambda }^{2}=\sum ({\rm{G}}-1)/{\lambda }^{2}={(\Delta \rho )}^{2}\phi (1-\phi ){\rm{\chi }}(G\,-\,1),$$

which finally contains only sample parameters, where G = G(ξ) depends on the scanning parameter ξ.

## Results

**Figure 7:**
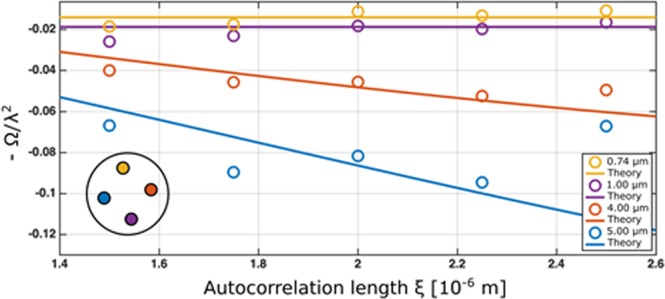
Wavelength corrected negative dark field coefficient −Ω/λ^2^ data extracted from the tomographic volumes (points) and corresponding model calculation Σ(G − 1)/λ^2^ (lines) in dependence on the real space correlation length ξ probed by the measurements.

The evolution of Eq. 6 describing the scattering structures as a function of the correlation length ξ is plotted in Fig. 7 for each of the four samples of colloidal suspensions in the cylindrical holes of the tomography sample. Each data point is an average over the respective uniform cylindrical sample volume. The plotted measurement values are complemented by continuous curves representing the expected theoretical results for the four samples, which were prepared with well-known reference particle radii, scattering length density contrast and concentrations. For the projected density correlation function we use the isolated hard sphere model as previously used for the description of this kind of sample^13^. In general good agreement was found when the design values of sample concentration, scattering density contrast and particle radius were used. One exception is the concentration parameter used for the 5 μm particles, which had to be increased by a factor 1.8 in order to match the data. However, when regarding the DFI projection image in Fig. 4, it can be seen that the respective particles concentrate towards the top, implying a higher particle density in the considered volume. When considering a deviation of the H_2_0/D_2_0 mixture in this sample, which could could increase particle buoyancy, such deviation could additionally contribute to an increased contrast.

The results for the 0.74 μm and the 1 μm particles are characterized by their slope of zero, which can easily be understood by considering that G becomes zero beyond the maximum structure size present in the sample. Correspondingly, the remaining signal is described solely by the product of scattering length density contrast, volume fractions and maximum structure size. As the former are equivalent for the two samples the ratio between their values coincides with the ratio of the particle sizes. Correspondingly the scattering of the larger colloids, 5.1 μm and 4 μm is stronger, despite their curves are still falling, because the correlation length probed has not reached their maximum structure size, i.e. the value of their diameters yet. The decrease of the depicted values agrees well with the trend of the model calculations. Despite the attempts to avoid dark-field signal saturation, the strong deviation of the last point for the 5 μm particles indicates that signal saturation causes this artefact. The increased volume fraction discussed above is suited to explain this occurrence.

## Conclusion

We presented clear experimental evidence for the potential of quantitative dark-field imaging extended to 3D by tomography. Quantitative characterization of heterogeneous microstructures in 3D opens up possibilities beyond current investigations limited to 2D implying the assumption of homogeneity in the third dimension, i.e. through the sample thickness. We presented the capability of structural characterization with 3D spatial resolution of the analyzed volume by matching model data of dispersed micro particles with corresponding experimental data, thus also observing deviations from the reference model. We were able to explicitly distinguish solutions of particles with different diameters distributed in the reconstructed 3D volume. We reached a sensitivity that allowed us to differentiate between particle sizes different by 260 nm only in the case of the 0.74 μm and 1 μm and to straightforwardly quantify this difference. While the accessible correlation length scale was limited to a range from 1.5 μm to 2.5 μm, which is shorter than what has been achieved earlier^15^ it was still sufficient for our proof of principle experiment to study particle reference systems of particles of 0.74 μm, 1 μm, 4.1 μm and 5 μm diameter.

The utilized sample material has before served for the proof of principle and validation of quantitative DFI in 2D^13^ which we here successfully extended to 3D.

We presented here a variation of the correlation length ξ by means of a wavelength scan, which is a promising possibility for future 3D experiments in particular at the new generation of powerful pulsed neutron sources, such as J-Parc, ISIS, and in future ESS. The possibility of measuring a multitude of wavelengths simultaneously will significantly decrease the necessary exposure times for quantitative DFI tomography and thus enable a large range of efficient applications.
